# Modulating the human gut microbiome and health markers through kombucha consumption: a controlled clinical study

**DOI:** 10.1038/s41598-024-80281-w

**Published:** 2024-12-30

**Authors:** Gertrude Ecklu-Mensah, Rachel Miller, Maria Gjerstad Maseng, Vienna Hawes, Denise Hinz, Cheryl Kim, Jack A. Gilbert

**Affiliations:** 1 Department of Pediatrics and Scripps Institution of Oceanography, University of California San Diego, 9500 Gilman Drive, La Jolla, CA 92093 USA; 2 Department of Pediatrics, University of California San Diego, Rady’s Children Hospital, La Jolla, CA USA; 3 Institute of Clinical Medicine, Faculty of Medicine, University of Oslo, Oslo, Norway; 4 Dep. of Gastroenterology, Oslo University Hospital, Oslo, Norway; 5 Bio-Me, Oslo, Norway; 6 La Jolla Institute for Immunology (LJI), La Jolla, CA USA

**Keywords:** Diet, Fermented foods, Gut microbiome, Serum cytokines, Immunology, Microbiology, Biomarkers, Health care

## Abstract

Fermented foods are becoming more popular due to their purported links to metabolic health and the gut microbiome. However, direct clinical evidence for the health claims is lacking. Here, we describe an eight-week clinical trial that explored the effects of a four-week kombucha supplement in healthy individuals consuming a Western diet, randomized into the kombucha (*n* = 16) or control (*n* = 8) group. We collected longitudinal stool and blood samples to profile the human microbiome and inflammation markers. We did not observe significant changes in either biochemical parameters or levels of circulating markers of inflammation across the entire cohort. However, paired analysis between baseline and end of intervention time points within kombucha or control groups revealed increases in fasting insulin and in HOMA-IR in the kombucha group whereas reductions in HDL cholesterol were associated with the control group. Shotgun metagenomic analysis revealed the relative abundance of *Weizmannia*, a kombucha-enriched probiotic and several SCFA producing taxa to be overrepresented in consumers at the end of the intervention. Collectively, in our healthy cohort consuming a Western diet, a short-term kombucha intervention induced modest impacts on human gut microbiome composition and biochemical parameters, which may be attributed to relatively small number of participants and the extensive inter-participant variability.

## Introduction

Dietary strategies are recognized as significant modulators of the composition and function of the gut microbiome, which, in turn, can influence various aspects of human physiology, including metabolism. This interplay offers therapeutic promise for addressing diet-driven metabolic disorders^1,2^. Fermented foods containing bioactive compounds have garnered widespread interest and increasing consumer demand, particularly among modernized, industrialized populations, primarily driven by their perceived health-promoting benefits to minimize the impact of western diets^3–7^. Observational and limited human dietary intervention studies have indicated associations between the consumption of diets rich in fermented foods and reduced disease risk, enhanced longevity, and improved quality of life^4,5,8–10^. For example, a systematic review and meta-analysis on the effect of fermented dairy products on cardiometabolic health revealed an association between fermented milk consumption and a reduced risk of type 2 diabetes^11^. Also, a 2021 human dietary intervention study demonstrated that high consumption of fermented food increased microbiome diversity and correlated with improvements in several serum markers of inflammation, indicating that potential probiotic-like microorganisms in fermented foods may confer health benefits to humans^12^.

Dairy-based fermented foods have been more extensively studied for their health benefits^13,14^, but other fermented foods, such as fermented tea beverages have been under explored. Kombucha is a non-alcoholic or low-alcohol tea-based beverage enriched with prebiotic compounds, acetic- and lactic-acid bacteria and yeast^3,15^. Interactions between bacteria and yeast species can lead to the generation of a wide variety of metabolites with functional bioactivities, such as organic acids, vitamins, and phenolic compounds, which could potentially influence the gut microbiome and overall health^2,6,15,16^. Several studies in animal models have suggested that intake of microbially-rich kombucha may offer potential therapeutic benefits, such as anti-hyperglycemic, anti-oxidant and anti-inflammatory effects, and changes in the gut microbiota that are associated with improved metabolic health^16–22^. For instance, Jung et al. demonstrated that increased relative abundance of fecal *Lactobacillus* and a concurrent reduction in *Allobacullum, Turibacter and Clostridium* relative abundances correlated with suppression of liver fat accumulation and the mitigation of nonalcoholic fatty liver disease following kombucha administration in experimental mice^17^. In another study, a four-week kombucha intervention in type 2 diabetes-induced mice reduced hyperglycemia and improved type 2 diabetes outcome through gut microbiota changes resulting in elevated proportional abundance of short chain fatty acids producing bacteria and decreased abundance of pathogenic bacteria^22^. Although, the mechanisms underpinning the observed effects have not been rigorously investigated, they are likely to occur through multiple inter-connected processes that include microbial food metabolites, the gut microbial ecosystem, and the host.

While promising results have been observed in animal models, direct evidence supporting the health conferring immune and metabolic health benefits of kombucha in human population remain sparse. Only two recently published controlled clinical trials have described the health benefits of kombucha consumption in human participants^23,24^. For instance, in the Mendelson and colleagues study, consumption of kombucha for four weeks in adults with type 2 diabetes mellitus led to a significant fasting blood glucose reduction in comparison with baseline, a finding not observed in the placebo treated group. Other animal model studies suggest that kombucha has immunomodulatory properties, positively influencing inflammation cytokines expression^21,25,26^. Therefore, we reasoned that changes in markers of inflammation could be an indication that kombucha supplementation could beneficially impact the immune system of participants. However, none of the studies have directly evaluated the effects of kombucha intake on shifts in the gut microbiome. To drive the development of microbiota-directed interventions, human studies are necessary to understand the translational significance of pre-clinical findings and the potential benefit of kombucha on gut microbiome composition and overall human health.

Herein, we investigated the impact of regular consumption of kombucha for four weeks on markers of inflammation and composition of the gut microbiome of human adult participants consuming Western diets using metagenomic sequencing, biochemical and immune profiling. We showed that the kombucha supplement did not alter biochemical and inflammation profiles. Subtle changes in microbiota composition, driven by the enrichment of SCFA producing microbes including the kombucha-associated probiotic, *Weizmannia coagulans* were observed post-kombucha intervention. Our results reflect the relatively small number of participants, short study duration and the extensive inter-individual heterogeneity.

## Results

### Free living participants successfully completed kombucha intervention.

To determine the effect of kombucha consumption on free-living adults on Western diets, we recruited participants for an eight-week randomized, controlled study. Of over 100 individuals initially expressing interest, 30 eligible individuals maintained interest after learning about all the study requirements and agreed to be enrolled in the trial. Participants were randomized (2:1) into the kombucha group (n = 20) or control group (n = 10). The primary outcome was change in microbiome composition at the end of the kombucha intervention period, while the secondary outcome was change in metabolic and immune markers after kombucha intervention.

**Fig. 1:**
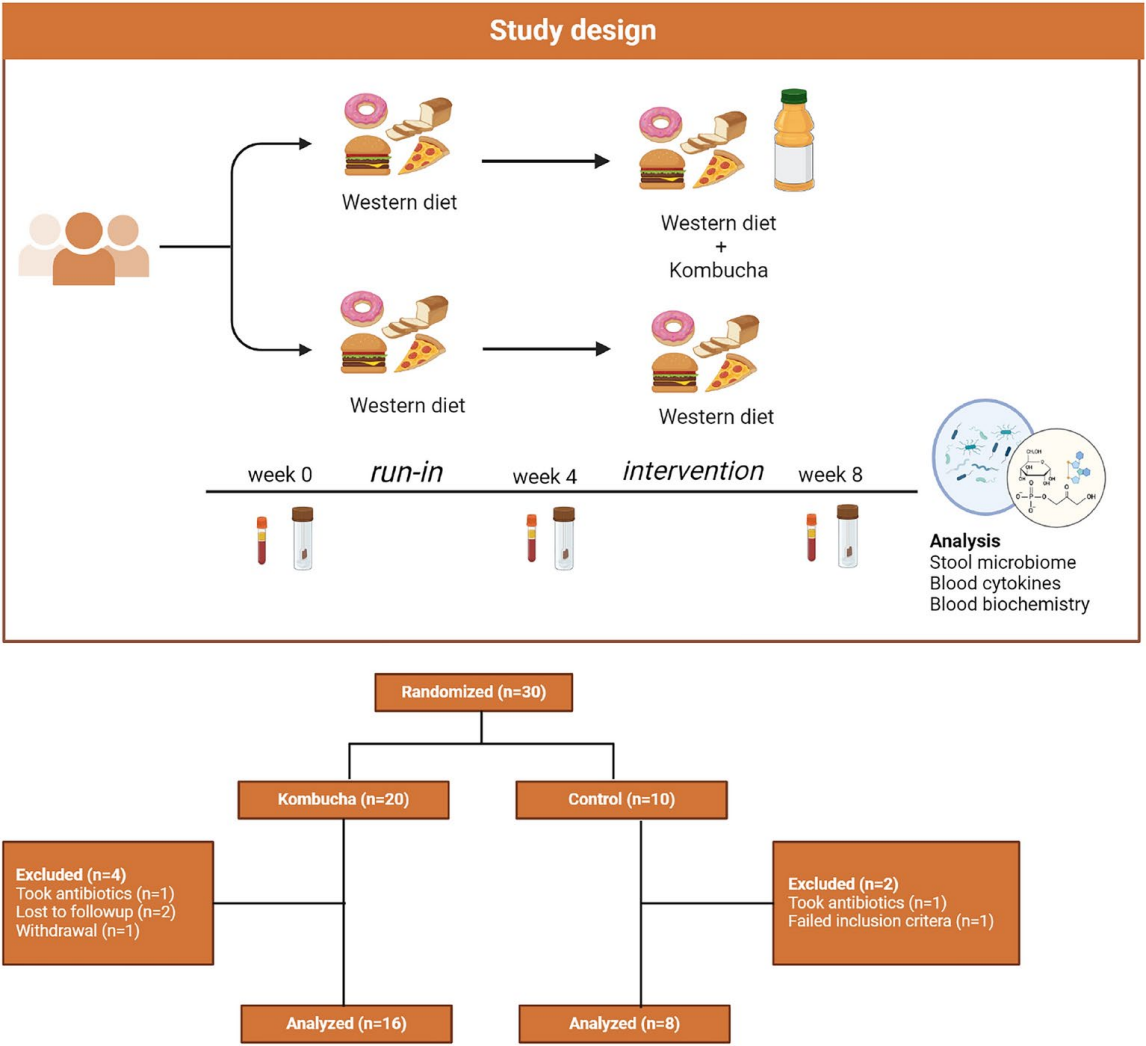
Flow diagram of study design, enrollment and allocation.

At the end of the trial, we had two participants, one in each group, taking antibiotics along the study time, one individual with higher HbA1c (> 5.7%), two participants lost to follow up without specific reasons and one individual who experienced severe bloating during the intervention and had to withdraw from the study (Fig. 1). These participants were excluded from the analysis, and hence the number of participants was reduced to 24 (16 intervention, 8 controls), o\;btained from 11 male and 13 female participants. All participants provided stool and blood samples at baseline (timepoint 1), four weeks after baseline collection (timepoint 2) and at the end of the four-week intervention period (timepoint 3) in which participants in the kombucha group took two servings of kombucha (16 oz in total per day). Approximately 96% self-reported completing the intervention. Self-reported gastrointestinal discomforts throughout the study are shown in Supplementary Table 2. Diarrhea and bloating were the most common discomforts reported by the kombucha group (31.25%) compared with 12.5% reported for the control group. Abdominal pain was frequently reported by the control group (25%), whereas the frequency in the kombucha group was about 12.5%.

### Kombucha intervention did not influence on anthropometric, metabolic or immune parameters.

We analyzed the anthropometric measurements and as shown in Table 1, we did not find any statistically significant difference between the control and the kombucha groups at any time point of the study. The secondary outcome of the study was change in metabolic health parameters in participants consuming kombucha from time point 1(baseline) to end (time point 3) of intervention. There were no significant changes in diastolic blood pressure, systolic blood pressure, fasting glucose levels, triglycerides, HDL cholesterol, or waist circumference over time between groups (unpaired Wilcoxon test) except for baseline cholesterol levels where control participants had significantly higher levels (unpaired Wilcoxon test; p = 0.035; Table 1; Supplementary Table 3). Fasting insulin and LDL cholesterol, elevated levels of which are associated with deteriorating health also did not differ between time points 1 and 3 between groups (unpaired Wilcoxon test; Table 2). Within the kombucha group (*n* = 16), significant increases were observed for fasting insulin levels (paired Wilcoxon test; p = 0.021) and HOMA-IR (paired Wilcoxon test; p = 0.021) over time (Supplementary Table 3). A significant decrease in HDL cholesterol levels (paired Wilcoxon test; p = 0.042) and marginal increase in creatinine levels (paired Wilcoxon test; p = 0.050) were observed for the control group (n = 8) over time (Supplementary Table 3).

**Table 1:** Baseline biochemical and anthropometric characteristics of participants.

Variable	Baseline		p-value
	Kombucha (n = 16)	Control (n = 8)	
BMI (kg/m2)	23.70(4.28)	23.63(4.61)	0.78
Age(years)	25.50(6.50)	26.00 (9.50)	1.00
Anion gap (mmol/L)	9.00(2.00)	8.50(1.50)	0.34
Bicarbonate (mmol/L)	27.00(3.25)	26.50(2.50)	0.78
BUN (mg/dL)	10.50(4.75)	10.50(7.00)	0.98
Calcium (mg/dL)	9.70(0.83)	9.65(0.23)	1.00
Chloride (mmol/L)	103.00(1.25)	103.50(1.25)	0.85
Cholesterol (mg/dL)	155.00(25.75)	186.50(34.75)	0.04
Creatinine (mg/dL)	0.76(0.17)	0.82(0.11)	0.81
Diastolic BP (mmHg)	70.00(14.38)	66.75(15.50)	1.00
Systolic BP (mmHg)	116.25(12.13)	111.00(17.50)	0.43
EGFR (mL/min)	61.00(0.00)	61.00(0.00)	0.96
Fasting insulin (uu/mL)	6.15(2.98)	7.40(5.38)	0.65
Glucose (mg/dL)	90.50(12.25)	92.00(3.50)	0.67
HbA1c (%)	5.05(0.30)	5.10(0.30)	0.95
HDL-C (mg/dL)	56.50(13.75)	63.00(20.50)	0.48
Height (cm)	172.20(13.59)	178.73(25.65)	0.44
Hip (cm)	96.45(10.10)	103.5(12.31)	0.30
HOMA-IR	1.39(0.89)	1.69(1.27)	0.60
LDL cal (mg/dL)	91.50(15.50)	103.00(24.50)	0.19
Non HDL-C (mg/dL)	100.50(14.50)	121.50(38.25)	0.12
Potassium (mmol/L)	4.35(0.48)	4.30(0.40)	1.00
Pulse	63.50(10.38)	69.25(12.13)	0.52
Respiration	16.00(0.50)	16.00(1.00)	0.77
Sex, female (%)	10.00(62.50)	3.00(37.50)	na
Sodium (mmol/L)	116.25(12.13)	140.50(1.75)	0.46
Temperature (oC)	36.65(0.23)	36.65(0.15)	0.90
Triglyceride (mg/dL)	59.00(25.25)	69.50(20.00)	0.52
Waist (cm)	84.68(7.36)	83.75(14.65)	0.58
Weight (kg)	71.13(13.29)	78.35(18.58)	0.41

Data are shown as median with interquartile range. Sex is shown as number of participants (with the percentage in brackets).

*BP* blood pressure, DBP, *BUN* Blood urea nitrogen, *HDL-C* high-density lipoprotein cholesterol, *LDL-C* low-density lipoprotein cholesterol, *BMI* body mass index, *na* not analyzed.

*p*-values, as determined by 2-sided Wilcoxon test, between groups are shown.

**Table 2:** Time point 3 biochemical and anthropometric characteristics of participants.

Variable	Final		P value
	Kombucha (n = 16)	Control (n = 8)	
BMI (kg/m2)	23.59(3.93)	23.63(4.98)	0.88
Age(years)	25.50(6.50)	26.00(9.50)	1.00
Anion gap (mmol/L)	10.00(1.25)	10.00(1.50)	0.45
Bicarbonate (mmol/L)	26.00(3.00)	26.00(1.50)	1.00
BUN (mg/dL)	13.50(6.00)	9.00(1.75)	0.08
Calcium (mg/dL)	9.60(0.33)	9.60(0.30)	0.42
Chloride (mmol/L)	103.00(2.00)	103.00(1.50)	0.83
Cholesterol (mg/dL)	163.00(25.75)	195.00(64.75)	0.28
Creatinine (mg/dL)	0.76(0.16)	0.85(0.19)	0.48
Diastolic BP (mmHg)	72.25(16)	71.00(8.38)	0.95
Systolic BP (mmHg)	118.00(12.13)	114.50(11.88)	0.34
EGFR (mL/min)	61.00(0.00)	61.00(0.00)	0.78
Fasting insulin (uu/mL)	9.30(4.83)	8.60(2.20)	0.76
Glucose (mg/dL)	91.50(7.25)	91.50(11.50)	0.83
HbA1c (%)	5.30(0.33)	5.20(0.15)	0.35
HDL-C (mg/dL)	58.00(14.25)	54.00(21.25)	0.48
Height (cm)	172.03(13.94)	179.18(24.79)	0.37
Hip (cm)	96.36(8.51)	101.20(7.03)	0.12
HOMA-IR	2.01(1.29)	1.82(0.53)	0.88
LDL cal (mg/dL)	96.50(25.25)	111.00(47.50)	0.48
Non HDL-C (mg/dL)	109.50(31.75)	131.50(53.75)	0.26
Potassium (mmol/lL)	4.35(0.33)	4.25(0.23)	0.56
Pulse	63.50(16.00)	65.50(14.63)	0.50
Respiration	15.75(3.00)	15.75(2.00)	0.70
Sex, female (%)	10.00(62.50)	3.00(37.50)	na
Sodium (mmol/L)	139.00(2.00)	138.50(2.25)	0.78
Temperature (oC)	36.66(0.23)	36.60(0.11)	0.14
Triglyceride (mg/dL)	57.00(38.00)	72.00(29.25)	0.36
Waist (cm)	81.55(8.33)	81.88(13.09)	0.69
Weight (kg)	71.23(12.14)	77.80(18.69)	0.43

Data are shown as median with interquartile range. Sex is shown as number of participants (with the percentage in brackets).

*BP* blood pressure, DBP, *BUN* Blood urea nitrogen, *HDL-C* high-density lipoprotein cholesterol, *LDL-C* low-density lipoprotein cholesterol, *BMI* body mass index, *na* not analyzed.

*p*-values, as determined by 2-sided Wilcoxon test, between groups are shown.

**Fig. 2:**
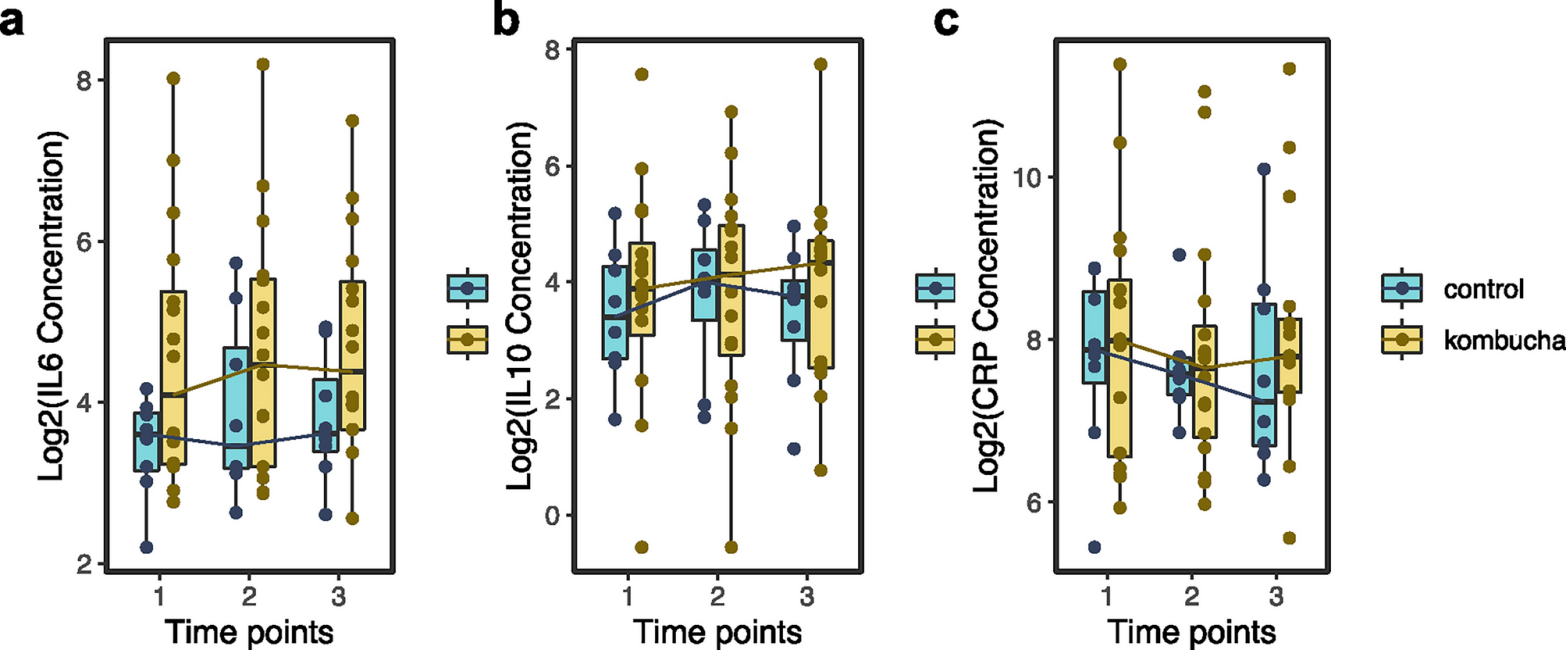
Differences in serum inflammation marker concentrations between the kombucha and control groups over time. IL6, interleukin 6; IL10, interleukin10; CRP, c-reactive protein. There were no significant differences within or between groups at each time point as assessed by Wilcoxon test (p > 0.05).

Despite the lack of generalized changes in several metabolic health parameters in the kombucha group relative to the control group, we wondered whether there were detectable changes in the immune system of participants in response to kombucha consumption. The sera of participants were analyzed for three different circulating cytokines (IL10, IL6 and CRP). No significant changes in immune features from baseline to end of intervention within participants were observed in either the kombucha or control groups (Fig. 2). Direct comparison of control and kombucha arms at the end of the intervention also did not reveal any differences indicating that the kombucha intervention did not influence inflammation in our cohort.

### Kombucha consumption marginally shifts gut microbiota and functional profiles.

We hypothesized that any effect of kombucha consumption would be captured in a microbiome-induced shift and demonstrable in stool samples. To characterize the effect of kombucha consumption on the microbiota of adults, fecal samples were subjected to shotgun sequencing and Operational Genomic Units (OGUs) were assigned by the Web of Life reference database^27^. After quality filtering, a total of 2,369,069,908 reads with a median of 9,647,881 reads and 2,128 taxa were generated from 200 samples (2–3 samples per 3 time points) from 24 unique participants. Since each participant collected 2–3 fecal samples from consecutive days at each time points, these replicates were merged for analyses. Merged replicates generated a median of 28,353,277 sequences from 72 samples, which was rarefied to equal depth of 2,959,909 reads per sample for diversity measures.

**Fig. 3:**
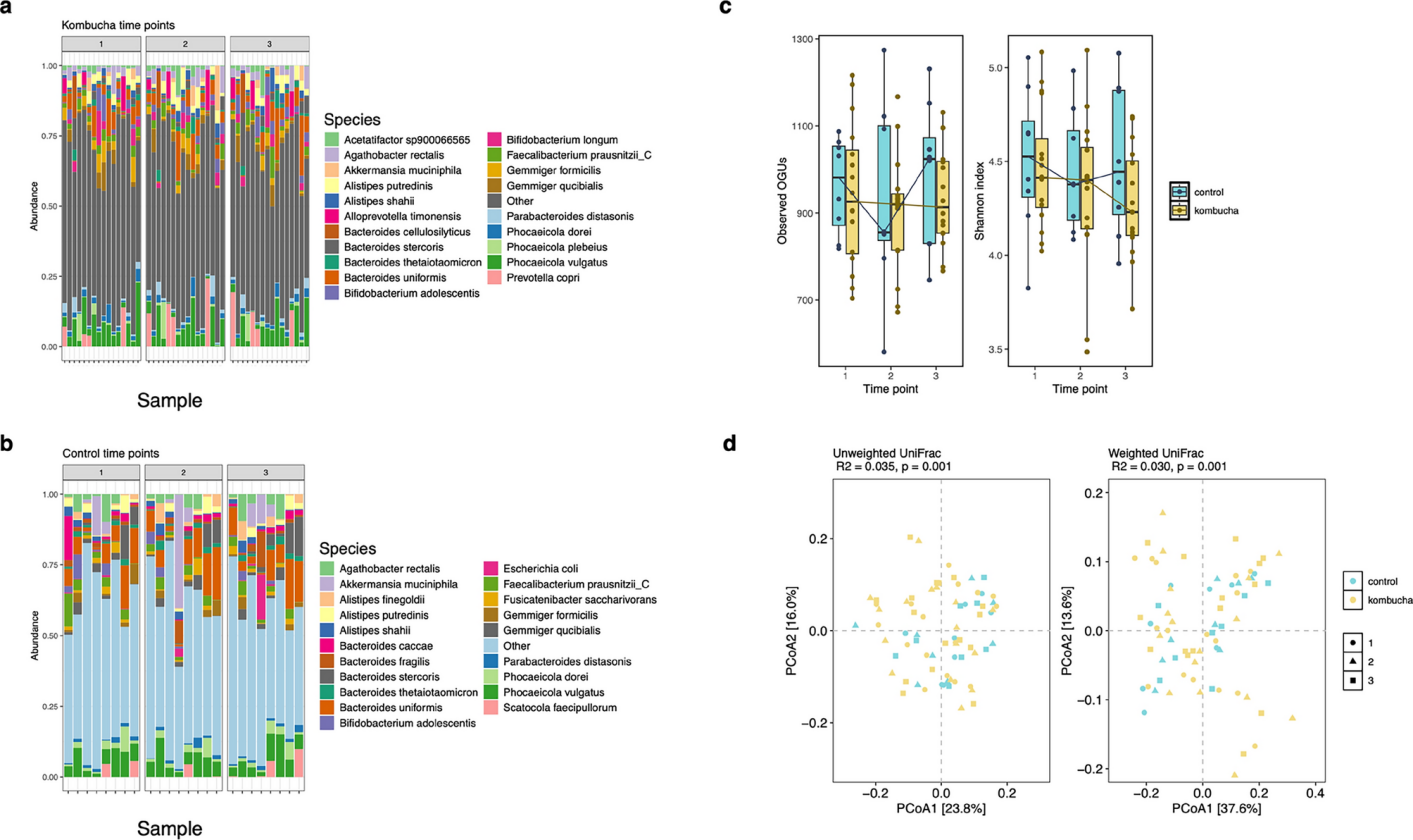
Changes in gut microbiome between kombucha and control groups over time. (**a**). The relative abundance of top 20 bacterial species in gut microbiome of control samples. (**b**). The relative abundance of top 20 bacterial species in gut microbiome of intervention samples. Each vertical bar represents an individual sample. (**c**). Observed OGUs and Shannon diversity for control and intervention groups at each time point. Shannon diversity significantly decreased between time point 1 and time point 3 for the kombucha group (Wilcoxon test). Boxplots denote the interquartile range (IQR) between the first and third quartiles, and the horizontal line defines the median. (**d**). Principal coordinate analysis (PCoA) of gut microbiota community structure measured by unweighted uniFrac and weighted uniFrac distances and calculated for OGU level composition. Composition of gut microbiota differed significantly between control and kombucha group with all time points included. Significance testing was performed with permutational analysis of variance (PERMANOVA; permutation *n* = 999). OGUs, operational genomic units.

Compositional analysis showed that the gut microbiota of participants in the kombucha and control groups across all time points was dominated by 5 major phyla, namely Bacteroidota (47%), Firmicutes_A (30.90%), Actinobacteriota (5.18%), Proteobacteria (7.53%) and Verrucomicrobiota (6.00%), contributing over 96% of the total composition (Supplementary Fig. 1a). The top 10 prevalent genera were *Bacteroides, Phocaeicola, Akkermansia, Escherichia, Alistipes, Prevotella, Bifidobacterium, Gemmiger, Agathobacter* and *Faecalibacterium* (Supplementary Fig. 1b)*.* In Figs. 3a&b which captures the relative abundance of the top 20 species in each group, *Prevotella* was associated with the kombucha group, whereas *Escherichia* was observed in the control group.

Alpha (α) diversity was measured using the Shannon Index and Observed OGUs with host id as a random effect. There was no significant difference in Observed OGUs between the intervention and control groups over time (Fig. 3c; LME; p > 0.05). However, we noted a significant reduction in the Shannon Index from time point 1 to the end of study (LME; p = 0.029) and a trend towards significance from time point 1 to time point 2 (LME; p = 0.05). At the end of intervention, kombucha and control groups did not differ significantly in any of the measured α-diversity indices (time point 3; p > 0.05; unpaired Wilcoxon test).

Within groups, Shannon Index decreased from time point 1 to time point 2 (LME; p = 0.043) and also from time point 1 to timepoint 3 (LME; p = 0.006) with host id as random effect in the kombucha group, however significance remained only for time point 1 vs time point 3 (q = 0.016) after multiple testing correction. The observed decline in the Shannon Index suggests a potential marked shift among certain microbial species at the end of intervention, resulting in altered evenness in species distribution and decreased overall gut microbiota diversity. By contrast, there was no differences in microbiota α-diversity (Shannon and Observed) over time within the control group.

Next, we calculated three different beta diversity measures (Fig. 3d; Supplementary Tables 4–6) and found significant differences in overall composition between kombucha and control groups (unweighted uniFrac, PERMANOVA, R^2^ = 0.04, p = 0.001; weighted uniFrac, PERMANOVA, R^2^ = 0.03, p = 0.001; Bray Curtis, PERMANOVA, R^2^ = 0.04, p = 0.001) and significant time effects for weighted uniFrac distance (PERMANOVA, R^2^ = 0.01, p = 0.026) and a trend towards significance for Bray Curtis dissimilarity (PERMANOVA , R^2^ = 0.01, p = 0.066). Host effects accounted for most variance (PERMANOVA, R^2^ = 0.61–0.70, p = 0.001). Within the kombucha group, the microbiota composition differed significantly post-intervention compared to baseline (Bray Curtis, PERMANOVA, R^2^ = 0.01, p = 0.025; weighted uniFrac, PERMANOVA, R^2^ = 0.02, p = 0.034; Supplementary Tables 7,8). Together, our results suggest that while study participant accounted for most of the variation, time and kombucha intervention had modest but significant impacts on total microbiota community structure dissimilarity.

**Fig. 4:**
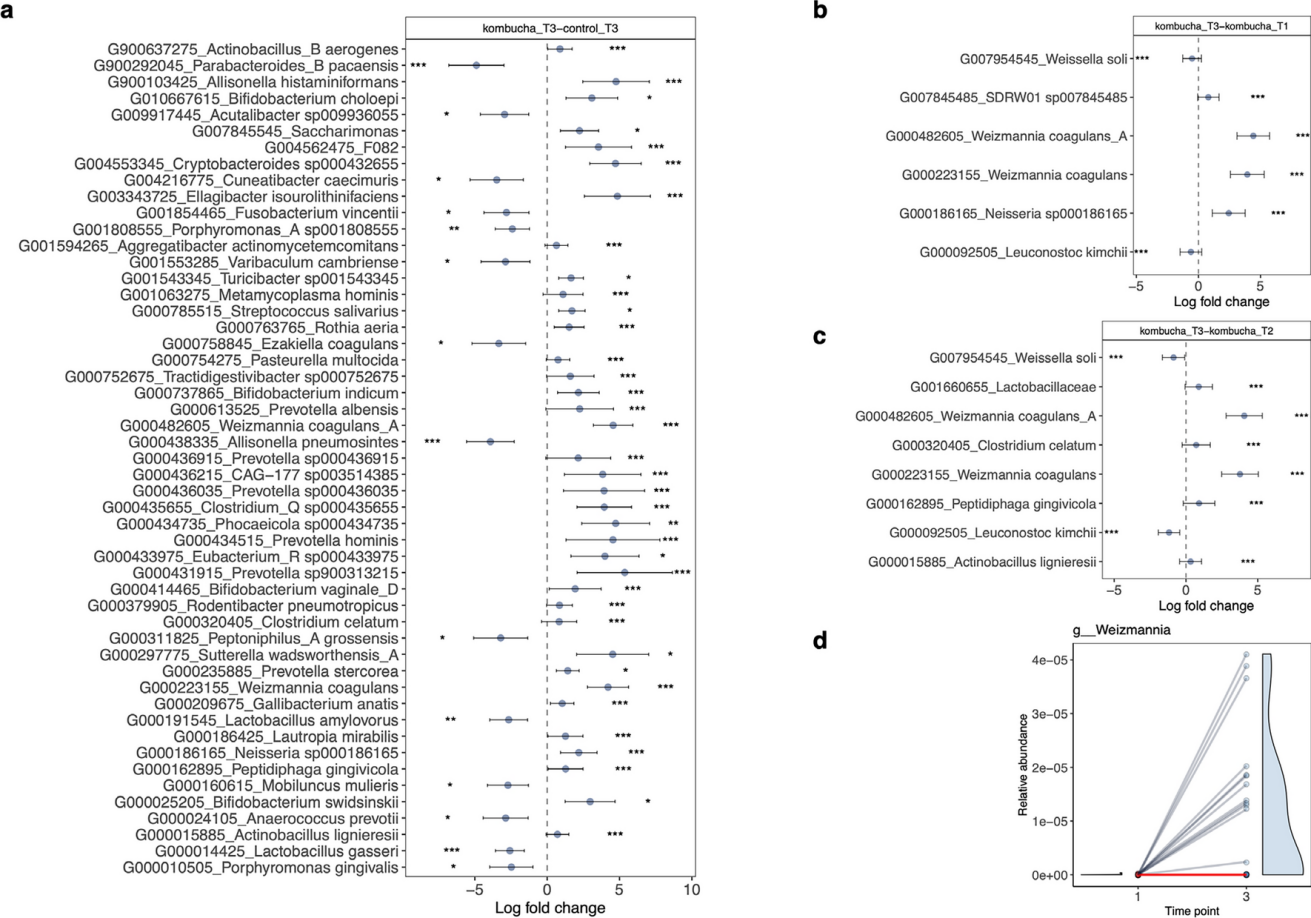
Microbial features associated with kombucha intervention. Differentially abundant features at the species level in gut microbiome samples in (**a**). control (*n* = 8) vs kombucha (*n* = 16) groups at the end of intervention (time point 3). (**b**). Kombucha microbiome samples before (time point 1) and after intervention. (**c**). Kombucha microbiome samples at time point 2 pre-intervention and after intervention. Colored dots represent the log-fold-change (effect size) with individual bacterial features showing significant effect sizes (*q* < 0.05). A positive log-fold-change indicates that a feature is more abundant in participants in the post-treatment kombucha group (Kombucha_T3), and a negative log-fold-change indicates a higher abundance in participants in the comparison group (control_T3, Kombucha_T1, Kombucha_T2). Whiskers represent 95% confidence interval (CI) calculated from the beta coefficient and standard errors estimated derived from the ANCOMBC (Analysis of compositions of microbiomes with bias correction) model. (**d**). Changes in the relative abundance of all members of the *Weizmannia* genus pre- (T1) and post- (T3) intervention in the kombucha group.

**Fig. 5:**
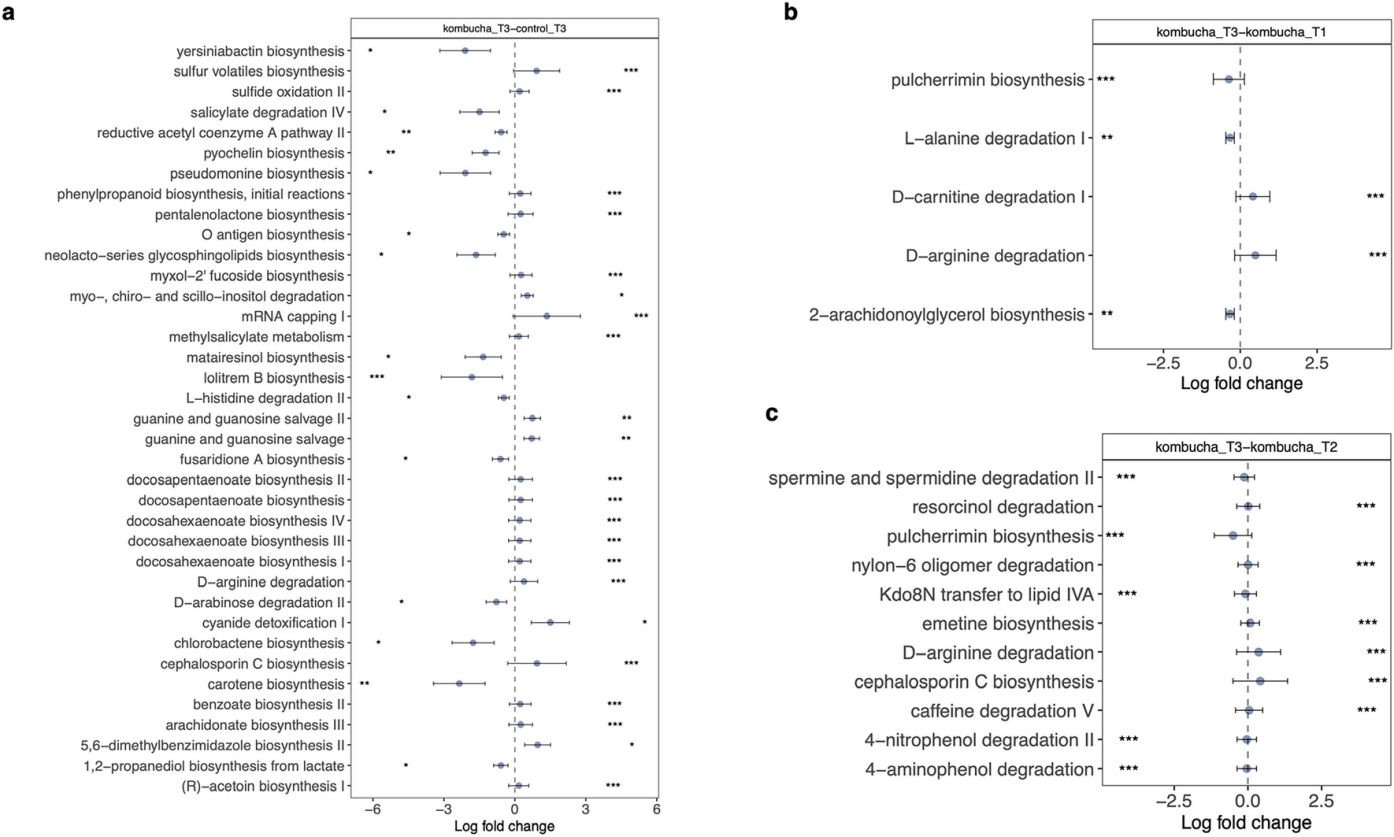
Microbial functions associated with kombucha intervention. Differentially abundant MetaCyc pathyways in gut microbiome samples in (**a**). control (*n* = 8) vs kombucha (*n* = 16) groups at the end of intervention (time point 3). (**b**). Kombucha samples before (time point 1) and after intervention. (**c**). Kombucha samples at time point 2 pre-intervention and after intervention. Colored dots represent the log-fold-change (effect size) with individual MetaCyc pathways showing significant effect sizes (*q* < 0.05). A positive log-fold-change indicates that a pathway is more abundant in participants in the post-treatment kombucha group (Kombucha_T3), and a negative log-fold-change indicates a higher abundance in participants in the comparison group (control_T3, Kombucha_T1, Kombucha_T2). Whiskers represent 95% confidence interval (CI) calculated from the beta coefficient and standard errors estimated derived from the ANCOMBC (Analysis of compositions of microbiomes with bias correction) model.

To examine whether specific microbial species were significantly different between the kombucha and control groups at the end of intervention, we ran the ANCOMBC algorithm for compositional differential abundance testing, which identifies genomic features characterizing the differences between two or more biological conditions^28^. Results from this analysis shown in Fig. 4, revealed that the microbiota of participants in the kombucha group were enriched in the relative proportions of several species including the kombucha-containing bacteria, *Weizmannia coagulans* compared with those of the control group at the end of intervention (Fig. 4a). Similarly, *Weizmannia coagulans* was differentially abundant among other species in the kombucha group at the end of intervention compared with baseline or time point 2 (Fig. 4b,d). Our observed results indicated that kombucha intervention resulted in detectable changes in the gut microbiota of consumers. In parallel with the shifts observed in the microbiota community composition across the kombucha and control groups, we observed shifts in metabolic functional pathways. Significant changes in the relative abundance of 37 MetaCyc pathways were found when comparing the kombucha and control groups at the end of intervention, 22 of which were enriched in the kombucha participants. Majority of the kombucha-enriched pathways were responsible for biosynthesis of nucleotide and nucleoside, biosynthetic pathways of small molecules such as cofactors, electron carriers, and vitamins, sulfur volatiles biosynthesis, cyanide detoxification, inositol degradation pathway. Conversely, kombucha consumption was linked with depletion in the relative proportions of pathways involved in secondary metabolites biosynthesis including carotene, siderophores (pyocherin, pseudomonine, yersiniabactin), and salicylate degradation pathway. Similarly, D-arginine degradation pathway was differentially abundant among others in the kombucha group at the end of intervention compared with baseline or time point 2 (Fig. 5b&c). Finally, no statistically significant evidence was found for correlations between OGUs that differed following 4 weeks of kombucha consumption and the significant biochemical parameters (fasting insulin, creatinine, HDL cholesterol, HOMA-IR) using Spearman’s correlation coefficient.

## Discussion

Here, we describe a randomized controlled human study examining the effects of a daily portion of kombucha dietary supplementation in free-living healthy individuals consuming a Western diet, combining high dimensional, longitudinal microbiome and markers of systemic inflammation profiling. We did not observe changes in biochemical parameters between kombucha consumers and controls, a finding consistent with previous randomized clinical trials in healthy cohorts^12,29^. While all participants were within the normal range for the measured health indicators, paired analysis between baseline and end of intervention for kombucha or control groups however, revealed increases in fasting insulin and HOMA-IR in kombucha group whereas reductions in HDL cholesterol were associated with the control group. Levels of circulating markers of inflammation were not significantly different between kombucha consumers and controls or within groups over time, suggesting that kombucha supplementation may have little impact on inflammation in healthy participants. Analysis of shotgun metagenomic sequencing data revealed that overall microbial diversity did not differ between kombucha consumers and controls, though marked differences in microbiota community composition and individualized microbiomes were observed. Within the kombucha group, microbiota diversity decreased modestly between baseline and end of intervention. Further, we found kombucha-containing *Weizmannia coagulans* to be enriched in consumers, suggesting that consumption of kombucha may trigger measurable shifts in the gut environment. Taken together, our findings suggest that in our healthy cohort, minor effects of kombucha intervention on human gut microbiota structure and biochemical parameters are observed, which may be attributed to relatively small number of participants and the extensive inter-individual variability.

Several studies have indicated that dietary interventions may cause profound changes in microbiota structure^12,30–32^. While in this study, a four week-daily kombucha consumption resulted in a minimal decrease in microbiota diversity, a finding which contrasts with the increased microbial diversity frequently associated with individuals consuming fermented foods^12,33^. The lack of large-scale microbial changes could partly be attributed to study factors, such as sample size or short duration of the study which might not have provided sufficient time for microbiota reshaping. Our study participants maintained a low fiber Western diet and it is possible that kombucha intervention on a background of Western diet has limited impact on microbial diversity in humans. Most studies have utilized a diverse mix of fermented foods^12,34–36^ in contrast to our study that relied on a single fermented food supplement approach and to our knowledge, this is the first study of the association between kombucha consumption and the human gut microbiome. Furthermore, existing studies vary in the quantity as well as the type of fermented food and duration of consumption, fermented food matrices, study designs, participant characteristics and health outcomes targeted. For example, increasing consumption of different fermented foods to six servings per day over 10 weeks in an intervention study elicited changes in gut microbial diversity^12^. By contrast, participants consumed 2 servings of kombucha for four weeks, suggesting that in our short intervention study, this fermented food threshold or magnitude may have been insufficient to induce broad microbial remodeling as observed previously^12^. Nevertheless, our findings are in line with other studies that have reported no changes to microbiota diversity as a result of fermented food intake in comparison with controls or pre-intervention state^29,34–37^. For instance, a randomized, double-blind, controlled intervention study evaluated the effect of daily consumption of two doses of a multi-strain fermented milk product for 4 weeks on the gut microbiome. The authors reported no changes in microbial alpha diversity after the intervention with the fermented product^37^. Similarly, habitual consumption of plant based fermented foods did not alter alpha diversity in relation to non-consumers^36^.

While overall shifts in microbial diversity were unaffected, our study observed that the composition of the gut microbiome was impacted by kombucha supplementation. We detected significantly altered differential abundance of selected taxa between consumers and controls at the end of intervention, a pattern consistent with earlier studies^12,29,33,35,36^. Specifically, we observed the enrichment of 36 species and the concurrent depletion of 14 taxa associated with kombucha consumers. We found the enrichment of short-chain fatty acids producing taxa including several species within the genus *Prevotella* and *Bifidobacterium*, *Eubacterium*, *Clostridium*, and *Ellagibacter isourolithinifaciens* and bacteria with less-favorable effects such as *Neiserria*. Importantly, of the microbial species present in the kombucha brand consumed, *Weizmannia coagulans* (*Bacillus coagulans*) was more frequently detected in the gut microbiome samples of consumers. Indeed, within the kombucha group, changes in community composition post-kombucha intervention were largely driven by shifts in *Weizmania coagulans*. Additionally, we detected significant depletions in the relative abundance of *Parabacteroides*, *Allisonella*, *Lactobacillus* and *Fusobacterium* among others in the kombucha cohort.

The dominance of *Weizmannia coagulans* in the kombucha consumers indicates that within four weeks of kombucha administration, the gut microbiome composition is characterized by an increase in bacteria ingested from the product. Our finding aligns with a prior study on the kombucha microbiota that identified *Weizmannia* as the most abundant bacterium present in the specific kombucha brand used in our study^38^. *Weizmannia coagulans* (*Bacillus coagulans*) is a Gram‐positive, lactic acid‐producer, non‐pathogenic and spore‐forming bacterium, widely used as a commercial probiotic in foods^39,40^. *Weizmannia coagulans* is a preferred probiotic due to its resistance to high temperature, high survival rate under harsh low‐oxygen environments of the gastrointestinal tract, stomach acids and bile salts as well as food processing^40,41^. *Weizmannia coagulans* has many known effects on digestive health, nutrient absorption, and human health through multiple mechanisms, including enzyme production, metabolite generation and modulation of the gut environment^42^. Its remarkable characteristics may explain its potential transient colonization in the gut following four-week kombucha consumption. Most claims about functional and health promoting properties related to kombucha are due to the high content of phenolic compounds present in the beverage^43–46^. Certain strains of *Weizmannia coagulans* can restructure the gut microbiota by increasing the abundance of intestinal transforming ellagic acid bacteria such as *Ellagibacter isourolithinifaciens* to facilitate the bioavailability of polyphenols^47,48^. In our study, we detected proportional increases of *Ellagibacter isourolithinifaciens*, after four weeks of kombucha consumption, known to be involved in dietary polyphenol metabolism in healthy individuals^49,50^. As our specific kombucha brand has been reported to be rich in polyphenols^38^, we speculate that kombucha consumption may have promoted the abundance of *Ellagibacter isourolithinifaciens* in the gut of consumers. Our results on the microbial functions suggest potential interactions between the gut microbiota and kombucha consumption. For instance, we observed enrichment in bacterial pathways for metabolic processes, including nucleotides and nucleosides biosynthesis, energy metabolism, and amino acids metabolism in the gut microbiota of kombucha consumers. Altogether, it is unclear if the observed changes are sufficient to elicit noticeable health benefits in our healthy participants, especially as we did not detect any profound differences in the biochemical parameters measured here.

While fermented foods have been associated with anti-inflammatory effects^10,12,14,51,52^, the absence of significant differences in the inflammatory profile of our study participants may be attributed to our limited sample size, short duration of the intervention and the quantity of fermented food consumed by participants. It is possible that other parameters of the immune system that are more malleable to dietary modulation in healthy individuals including white blood cell profile, T cells or a more comprehensive immune profiling^12,53^ could provide detailed insights into the immunomodulatory capabilities of kombucha.

Our exploratory investigation focused on a limited number of healthy participants over a short duration, potentially constraining our ability to discern significant impacts on microbial, biochemical and inflammation profiles. Moreover, the modest sample size contributed to intra-individual variations that surpassed the inter-individual heterogeneity in gut microbiota composition. Participants were recruited according to self-reported dietary information, which can be biased and inaccurate. We had no data on stool consistency measurements such as stool form and transit time, parameters known to alter the gut microbiota. This study did not profile the microbial and chemical constituents of the kombucha product used as they have been extensively characterized^38^. We acknowledge that batch-to-batch variations within the same kombucha product could exist, potentially confounding our findings. However, our results clearly indicate that the previously identified dominant bacterium in the kombucha microbiota^38^ was successfully detected in the fecal microbiota of consumers. Without microbial metabolites data, we were unable to explore possible mediators in response to kombucha intake in healthy participants. While our study suggests that kombucha supplementation may have influenced compositional shifts in the microbiome, we acknowledge the presence of other influencing factors, such as host genetics or nondietary behaviors. Moving forward, larger-scale randomized studies conducted over extended periods within clinical cohorts combining biomarkers of immune, metabolic, and endocrine function with microbiome data are essential to thoroughly explore the potential therapeutic utility of microbially-rich kombucha beverages in optimizing health outcomes.

In conclusion, a short term kombucha dietary intervention in healthy participants differentially influenced the composition of gut microbiota, enriching several SCFA producing taxa. However, these compositional changes did not correspond to broad shifts in biochemical or inflammation profiles, at least over this short-term intervention. Applying multi-omic approaches on larger sample size with longer study duration are needed to delineate the impact of kombucha consumption on gut microbiota modulation and its connection to human health and disease outcomes.

## Materials and methods

### Ethics statement

The research and related activities involving human subjects were approved by the Institutional Review Board at University of California, San Diego (IRB# 210,823) and registered at https://www.clinicaltrials.gov (NCT06484504; 03/07/2024). All research, experiments and data analysis were performed in accordance with the University of California guidelines and regulations. All participants provided informed written consent prior to enrollment and all research was performed in accordance with the federal guidelines and regulations and the Declaration of Helsinki.

### Study design

This was an eight-week, parallel, randomized clinical controlled trial where individuals were assigned 2:1 to either a kombucha intervention group or a non-kombucha control group (Fig. 1). There were 3 time points in the study, namely, time points 1 (baseline), 2 (four weeks from baseline) and 3 (eight weeks from baseline). Participants were randomized using a random number generator in Excel by a biostatistician who was not involved in the intervention or data collection. The clinic staff involved in the data collection were blinded to the study, however, it was not possible to blind enrolled participants. The first four weeks after time point 1 data completion, participants consumed a beige/Western diet (i.e. low-fiber, low polyphenol diet), thereafter, participants randomized into the intervention group consumed one bottle (two servings) of a 16 oz commercial kombucha daily for four weeks in addition to their beige diet, whereas control-randomized participants maintained their beige diet intake. During each study visit at time points 1, 2 and 3, fasting blood was drawn, while anthropometrics data, stool samples, questionnaires and three-day food records were collected. Compliance with dietary intervention and restrictions was assessed by asking whether participants consumed the kombucha product provided and whether they were able to limit or avoid foods listed in the provided guidelines (Supplementary Table 1). The study was designed as exploratory with the primary endpoint as changes in microbiota diversity after kombucha intervention, while secondary endpoints included changes in biochemical and immune markers (IL6, IL10, CRP). There were no significant changes to the methods after the trial began, except that body composition was not measured due to the instrument being inoperative throughout the study period. We relied on body weight determinations. Additionally, our secondary objective of measuring fecal metabolites was not determined due to an insufficient biomass in stool samples, a limitation that is addressed in the discussion.

### Eligibility and recruitment

Free-living participants were recruited from the San Diego County through online and paper advertisements as well as social media platforms (Facebook, LinkedIn, Twitter) and emails sent to the University of California (UC) San Diego community. We assessed 60 participants for eligibility where they answered questions on medical history, lifestyle habits, age, sex, antibiotic use, bowel movement, and diet, followed by a clinic visit for eligible participants. The primary inclusion criteria included the following: healthy adults aged 21–55 years, females must be non-menopausal, BMI between 18–29.9. Participants were excluded if they had a history of gastrointestinal surgery, diabetes mellitus on medications, or other serious medical condition, such as chronic hepatic or renal disease, bleeding disorder, congestive heart disease, chronic diarrhea disorders, myocardial infarction, coronary artery bypass graft, angioplasty within 6 months prior to screening, current diagnosis of uncontrolled hypertension (defined as systolic BP ≥ 160 mmHg, diastolic BP ≥ 95 mmHg), Other exclusion criteria included use of antibiotics or laxatives within the last three months, using prebiotics, probiotics, and/or any fiber supplements regularly and allergy or sensitivity to kombucha. Thirty participants passed the screening and were enrolled in the study.

### Product description

The investigated product used was a 16 oz of a commercial kombucha beverage, which according to the manufacturer contained a propriety blend of three probiotic strains. The concentrations of the probiotics *Bacillus coagulans* GBI-306086, *S*. *boulardii* and *Lactobacillus* bacterium were one billion, four billion and four billion organisms respectively and were used in the fermentation process. Other naturally occurring components in the product were lactic acid (100 mg), acetic acid (75 mg), glucuronic acid (1400 mg), gluconic acid (650 mg), with kiwi juice and ginger juice added as flavors. The microbial, chemical metabolic and nutritional profiles in the kombucha product have been assessed before^38^. Participants were instructed to take half portion of one bottle of the 16 oz kombucha twice a day, every day for the four weeks of intervention along with their Western diet.

### Sample collection and processing

Participants were requested to fast for at least 10 h prior to each clinic visit at the Altman Clinical Translational Research Institute, UC San Diego. Blood samples were collected from the antecubital vein into SST (serum separator tube) and EDTA vacutainers (Becton Dickinson, Franklin Lakes, NJ, USA), span at 1,200xg for 10 min for serum and plasma respectively, aliquoted, and stored at − 80 °C for downstream analysis. Blood collected for metabolic parameters were analyzed within two hours of collection at the CLIA-certified Center for Advanced Laboratory Medicine at UC San Diego. A wall-mounted stadiometer and an electronic scale were used to measure height and weight, respectively. Body mass index was calculated by dividing the weight (kg) by the height squared (m^2^). Waist circumference was measured midway between the lowest rib and the iliac crest using an anthropometric nonelastic tape, blood pressure, pulse and respiration were measured using the Mindray Accutorr 7 equipment.

Participants were provided sterile stool swab sampling kits with instructions on sample collection. Stool swab samples were collected at three different time points (1, 2, 3). Specifically, within three days prior to each study visit at time points 1 (baseline), 2 (four weeks) and 3 (eight weeks), participants collected three sets of stool swab samples which were preserved in ethanol and brought to each clinic visit. Stools samples were submitted at each clinic visit and then transferred to − 80 °C storage in the laboratory. At the end of the study, each participant was expected to submit nine stool samples in total.

### Blood biochemistry

Standard metabolic indicators were analyzed using Roche cobas® 8000 modular analyzer following standard procedures within two hours of blood collection. Homeostatic Model Assessment for Insulin Resistance (HOMA) index was calculated as insulin level (µU/ml) × glucose level (mmol/l)/22.5. Additional biochemical parameters evaluated included lipid levels (total cholesterol, HDL cholesterol, LDL cholesterol, triglyceride), creatinine level, and estimated glomerular filtration rate (eGFR).

### Inflammation markers

Data on inflammation markers were generated at the La Jolla Institute for Immunology. Briefly, the quantifications of IL-6, IL-10, and C- reactive protein (CRP) from serum were determined using bead-based LEGENDplex immunoassays as described by the manufacturer’s protocols (BioLegend, San Diego, CA). LEGENDplex assays were measured using a BD FACSCanto™ II flow cytometer (BD Biosciences), and data analyzed using LEGENDplex software (BioLegend) and R version 4.1.1.

### Metagenomic analysis

Nucleic acid extractions were performed at the UC San Diego Microbiome Core using previously published protocols^54^. Briefly fecal samples were randomly sorted, transferred to 96-well extraction plates and DNA was extracted using the MagMAX Microbiome Ultra Nucleic Acid Isolation Kit (Thermo Fisher Scientific, USA) and automated on KingFisher Flex robots (Thermo Fisher Scientific, USA). Blank controls and mock controls (ZymoBiomics) were included per extraction plate, which were carried through all downstream processing steps. Purified DNA was quantified using a PicoGreen fluorescence assay (Thermo Fisher Scientific, USA) and metagenomic libraries were prepared with KAPA HyperPlus kits (Roche Diagnostics, USA) according to the manufacturer’s instructions. Sequencing was performed on the Illumina NovaSeq 6000 sequencing platform with paired-end 150 bp cycles at the Institute for Genomic Medicine (IGM), UC San Diego. Raw metagenomic sequences were quality filtered and trimmed using fastp v 0.22^55^ and sequence reads mapping to the human genome and PhiX reads were filtered using minimap2 version 2.22^56,57^ and converted to FASTQ format using SAMtools version 1.15.1.^58^. Reads were aligned against the Web of Life reference database^27^ using Bowtie2^59^ and classified with the Woltka pipeline^60^ to generate a table of Operational Genomic Units (OGUs). Sequences matching the human reference genome and genomes with less than 0.5% coverage per sample and features with less than ten reads in the entire dataset were removed. Functional profiles were obtained using the Woltka pipeline^60^ and annotated using the MetaCyc database^61^.

### Statistical analyses

In the absence of a human intervention study on kombucha’s effect on gut microbiota, our sample size determination was based on a prior intervention study involving fermented soymilk’s impact on fecal microbiota^62^. In the study, the daily administration of fermented soymilk for two weeks to 10 healthy participants resulted in a significant increase in fecal *Bifidobacteria* and Lactobacilli copy numbers and a concurrent reduction in the copy numbers of Clostridia. Based on this data, with a sample size of 14 subjects in the treatment arm, we estimated > 90% power to detect a significant increase in Lactobacilli following the administration of a fermented food. Given the uncertainty regarding kombucha’s impact on intestinal microbiota relative to fermented soymilk, we anticipated that enrolling 20 participants (including potential dropouts) will provide sufficient power to detect changes in bacterial composition before and after kombucha consumption.

The biochemical and anthropometric data of the study participants were summarized using descriptive statistics, with continuous variables presented as median and interquartile range, while categorical variables were presented as number and percentages. Significant changes were evaluated from time point 1 to end of intervention (time point 3) by a paired Wilcoxon test (within groups) and unpaired Wilcoxon test (between control vs. kombucha groups). All microbiome samples were rarified to 2,959,909 reads, retaining 210/241 samples and alpha diversity measures quantified as Observed OGUs and Shannon Index were calculated from the OGU table using phyloseq^63^. Differences in alpha diversity over time were analyzed with linear mixed effects models (lme4 v 1.1–33) in R, where participants were treated as random effect while controlling for sex and age. Pairwise comparisons were assessed using the Tukey method. Beta diversity was determined using Bray Curtis dissimilarity, weighted and unweighted UniFrac distances^64^ generated in phyloseq and statistical differences were assessed with permutational multivariate analysis of variance (PERMANOVA) using the adonis2 function in the Vegan package. To assess longitudinal distance from baseline, sample dissimilarity between baseline and other time points for kombucha and control samples were calculated using the miaTime R package v0.1.15. Relative abundances at the phylum and species level were calculated using the unrarefied OGU table and presented as percentages. To determine differentially abundant features between control and intervention groups over time, the unrarefied OGU table was binned at the species level and the MetaCyc abundance table were used in the analysis of compositions of microbiomes with bias correction (ANCOMBC)^28^. Sex, BMI, and age were added as covariates in the ANCOMBC formula. All statistical tests were two-sided and *p* < 0.05 was deemed statistically significant. Adjustments for multiple comparisons (*q* values) were applied as indicated. Data analysis and figures were generated in R version 4.1.1.

## Supplementary Information

Supplementary Tables.

Supplementary Figures.

## Data Availability

The data supporting the findings of this study are available under restricted access due to the privacy regulations of our study participants. However, access can be obtained by request to the corresponding author.
