# Supplementary material for: Modulating the human gut microbiome and health markers through kombucha consumption: a controlled clinical study
Source: Sci Rep. 2024 Dec 30;14:31647. doi: 10.1038/s41598-024-80281-w (PMC11686376; doi:10.1038/s41598-024-80281-w)
Supplement: Supplementary file 1 — Supplementary Tables. [file 41598_2024_80281_MOESM1_ESM.docx]

Supplementary Table 1. High Fiber and Polyphenol Food/Beverage Lists

Participants are to follow a beige diet during the study period. A beige diet is defined by a low fiber (< 10 g daily) and low polyphenol diet (< 3 servings of polyphenol rich foods daily) during the study period. See the tables below for information regarding high fiber and polyphenol rich foods.

| **High Fiber Foods Items to Avoid/Limit**  Limit daily intake to <10 g of fiber | | **Serving** | **Grams** |
| --- | --- | --- | --- |
| **Vegetables** | Green beans, raw  Broccoli/Cauliflower, cooked  Turnip greens, cooked  Brussel sprouts, cooked  Potato (white) *with skin, baked  Sweet corn, cooked  Raw baby carrots  Raw medium carrot  Asparagus, cooked  Artichokes  Green peas  Lima beans  Acorn squash  Kale  Spinach, cooked  Spinach, raw  Eggplant, cooked  Cabbage, cooked  Cabbage, raw  Swiss Chard, cooked  Zucchini, cooked  Zucchini, raw  Bell peppers, raw  Bell peppers, cooked  Celery | 1 cup  1 cup  1 cup  1 cup  1 medium  1 cup  1 baby  1 medium  1 spear  ½ cup  1 cup  ½ cup  1 cup  1 cup  1 cup  1 cup  1 cup  1 cup  1 cup  1 cup  1 cup  1 cup  1 cup  1 cup  1 cup | 2.7 g  5 g  5 g  4 g  4 g  3.5 g 0.3 g 1.7 g  0.3 g  7 g  4 g  7 g  6 g  2.6 g  4 g  0.9 g 2.5 g 2.8 g  1.5 g  4 g  1.8g  1.1 g 2.5 g 1.1 g  1.6 g |
| **Fruits** | Coconut, raw/shredded  Blueberries  Dried apricot  Pear  Apple  Orange  Avocado, sliced  California Avocado, whole  Mango  Persimmon | 1 cup  1 cup  1 cup  1 medium  1 medium  1 small  1 cup  1 whole  1 cup  1 whole | 7 g  3.6 g  9 g  6 g  4.4 g  2.3 g  10 g  13 g  2.6 g  6 g |
| **Legumes/Nuts** | Almonds | 23 nuts | 3.5 g |

|  | Walnuts  Peanuts (including butter)  Pecans  Chia  Sunflower  Beans (navy, pinto, kidney, soy, chickpeas, lentils) | | ½ cup  ½ cup  ½ cup  1 tablespoon  1 tablespoon  1 cup | 4 g  6 g  5 g  10 g  3 g  15 g | |
| --- | --- | --- | --- | --- | --- |
| **Grains** | Wheat bran  Brown rice  Wild rice  Popcorn  Oatmeal  Granola  Shredded wheat  Quinoa  Bulgur  Barley  Lentils  Whole wheat pasta | | 1 cup  1 cup  1 cup  1 cup  1 cup  1 cup  1 cup  1 cup  1 cup  1 cup  ½ cup  1 cup | 25 g  3.5 g  3 g  6 g  4 g  10 g  20 g  4 g  10 g  10 g  8 g  6 g | |
| **High Polyphenol Foods Items to Avoid/Limit**  Limit daily intake to < 3 servings of polyphenol rich foods during the study period | | | | |  |
| **Spices/Herbs** | | Cloves Dried peppermint Star anise Cocoa powder | | |  |
| **Legumes/Nuts** | | Hazelnuts Walnuts Almonds Pecans Black Beans White beans | | |  |
| **Fruits** | | Blueberries Blackberries Strawberries Raspberries Plums Black Currants Cherries Apples (+juice) Pomegranate (+juice) | | |  |
| **Vegetables** | | Artichokes Chicory Red Onion Spinach Tempeh Beansprouts | | |  |
| **Beverages** | | Black tea Green tea Red wine | | |  |
| **Other** | | Dark chocolate Milk chocolate Yogurt | | |  |

| Supplementary Table 2. Self-reported gastrointestinal discomforts | | |
| --- | --- | --- |
|  | **kombucha (n=16)** | **control (n=8)** |
| Diarrhea | 5 (31.25 %) | 1 (12.5%) |
| Constipation | 2 (12.5%) | 1 (12.5%) |
| Bloating | 5 (31.25%) | 1 (12.5%) |
| Abdominal pain | 2 (12.5%) | 2 (25%) |
| Headache | 2 (12.5%) | 1 (12.5%) |
| Nausea | 1 (6.25%) | 1 (12.5%) |

Percentage expressed as number of participants who reported experiencing discomforts at least 3 or more days throughout the 8 week study period

Supplementary Table 3. Biochemical and anthropometric characteristics of participants

| **Variable** | **Baseline** | | **Final** | | **P-1** | **P-2** | **P-3** |
| --- | --- | --- | --- | --- | --- | --- | --- |
|  | **Kombucha (n=16)** | **Control (n=8)** | **Kombucha (n=16)** | **Control (n=8)** |  |  |  |
| BMI (kg/m2) | 23.70(4.28) | 23.63(4.61) | 23.59(3.93) | 23.63(4.98) | 0.78 | 0.84 | 0.29 |
| Age(years) | 25.50(6.50) | 26.00 (9.50) | 25.50(6.50) | 26.00(9.5) | 1 | na | na |
| Anion gap (mmol/L) | 9.00(2.00) | 8.50(1.50) | 10.00(1.25) | 10.00(1.50) | 0.34 | 0.40 | 0.43 |
| Bicarbonate (mmol/L) | 27.00(3.25) | 26.50(2.50) | 26.00(3.00) | 26.00(1.50) | 0.78 | 0.35 | 0.07 |
| BUN (mg/dL) | 10.50(4.75) | 10.50(7) | 13.50(6) | 9.00(1.75) | 0.98 | 0.11 | 0.57 |
| Calcium (mg/dL) | 9.70(0.83) | 9.65(0.23) | 9.60(0.33) | 9.60(0.3) | 1 | 0.50 | 0.18 |
| Chloride (mmol/L) | 103.00(1.25) | 103.50(1.25) | 103.00(2.00) | 103.00(1.50) | 0.85 | 0.34 | 0.58 |
| Cholesterol (mg/dL) | 155.00(25.75) | 186.50(34.75) | 163.00(25.75) | 195.00(64.75) | **0.04** | 0.83 | 0.33 |
| Creatinine (mg/dL) | 0.76(0.17) | 0.82(0.11) | 0.76(0.16) | 0.85(0.19) | 0.81 | **0.05** | 0.88 |
| Diastolic BP (mmHg) | 70.00(14.38) | 66.75(15.50) | 72.25(16.00) | 71.00(8.38) | 1 | 0.57 | 0.92 |
| Systolic BP (mmHg) | 116.25(12.13) | 111.00(17.5) | 118.00(12.13) | 114.50(11.88) | 0.43 | 0.94 | 0.81 |
| EGFR (mL/min) | 61.00(0) | 61.00(0) | 61.00(0) | 61.00(0) | 0.96 | 1 | 0.20 |
| Fasting insulin (uu/mL) | 6.15(2.98) | 7.40(5.38) | 9.30(4.83) | 8.60(2.20) | 0.65 | 0.95 | **0.02** |
| Glucose (mg/dL) | 90.50(12.25) | 92.00(3.50) | 91.50(7.25) | 91.50(11.50) | 0.67 | 0.67 | 0.47 |
| HbA1c (%) | 5.05(0.30) | 5.10(0.30) | 5.30(0.33) | 5.20(0.15) | 0.95 | 0.92 | 0.09 |
| HDL-C (mg/dL) | 56.50(13.75) | 63.00(20.50) | 58.00(14.25) | 54.00(21.25) | 0.48 | **0.042** | 0.78 |
| Height (cm) | 172.20(13.59) | 178.73(25.65) | 172.03(13.94) | 179.18(24.79) | 0.44 | 0.93 | 0.16 |
| Hip (cm) | 96.45(10.10) | 103.50(12.31) | 96.36(8.51) | 101.20(7.03) | 0.30 | 0.84 | 0.33 |
| HOMA-IR | 1.39(0.89) | 1.69(1.27) | 2.01(1.29) | 1.82(0.53) | 0.60 | 0.84 | **0.021** |
| LDL cal (mg/dL) | 91.50(15.50) | 103.00(24.50) | 96.50(25.25) | 111.00(47.50) | 0.19 | 0.58 | 0.31 |
| Non HDL-C (mg/dL) | 100.50(14.50) | 121.50(38.25) | 109.50(31.75) | 131.50(53.75) | 0.12 | 0.40 | 0.38 |
| Potassium (mmol/L) | 4.35(0.48) | 4.30(0.40) | 4.35(0.33) | 4.25(0.23) | 1 | 0.32 | 0.38 |
| Pulse | 63.5(10.38) | 69.25(12.13) | 63.50(16.00) | 65.50(14.63) | 0.52 | 0.40 | 0.86 |
| Respiration | 16(0.50) | 16.00(1.00) | 15.75(3.00) | 15.75(2.00) | 0.77 | 0.39 | 0.60 |
| Sex, female (%) | 10.00(62.50) | 3.30(37.50) | 10.00(62.50) | 3.30(37.50) | na | na | na |
| Sodium (mmol/L) | 116.25(12.13) | 140.50(1.75) | 139.00(2.00) | 138.50(2.25) | 0.46 | 0.32 | 0.14 |
| Temperature (oC) | 36.65(0.23) | 36.65(0.15) | 36.68(0.23) | 36.6(0.11) | 0.90 | 0.29 | 0.88 |
| Triglyceride (mg/dL) | 59.00(25.25) | 69.50(20.00) | 57.00(38.00) | 72.00(29.25) | 0.52 | 0.67 | 0.66 |
| Waist (cm) | 84.68(7.36) | 83.75(14.65) | 81.55(8.33) | 81.88(13.09) | 0.58 | 0.64 | 0.27 |
| Weight (kg) | 71.13(13.29) | 78.35(18.58) | 71.23(12.14) | 77.80(18.69) | 0.41 | 0.55 | 0.92 |

Data are shown as median with interquartile range. Sex is shown as number of participants (with the percentage in brackets). BP, blood pressure; DBP, BUN, Blood urea nitrogen; HDL-C, high-density lipoprotein cholesterol; LDL-C, low-density lipoprotein cholesterol; BMI, body mass index. *p*-values, as determined by 2-sided Wilcoxon test, between or within groups are shown. P-1, p value for control baseline versus kombucha baseline; P-2, p value for control baseline vs control final; P-3, p value for kombucha baseline vs kombucha final; na, not analyzed.


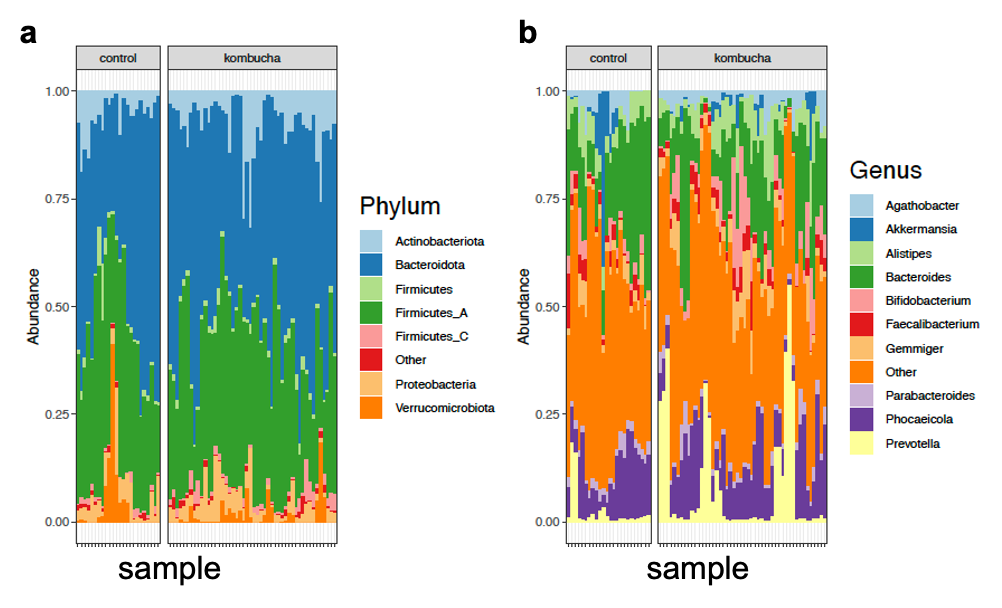


Supplementary Figure 1. Variation in gut microbiome between kombucha and control groups over time. a. The relative abundance of top seven bacterial phyla in gut microbiome of control and intervention samples. b. The relative abundance of top 10 bacterial genera in gut microbiome of control and intervention samples. Each vertical bar represents an individual sample.

| Supplementary Table 4. Permutational analysis of variance model overall results for weighted uniFrac matrix | | | | | |
| --- | --- | --- | --- | --- | --- |
|  | **Df** | **SumOfSqs** | **R2** | **F** | **Pr(>F)** |
| **Intervention** | 1 | 0.1033 | 0.02649 | 6.7389 | 0.001 *** |
| **Time** | 2 | 0.0645 | 0.01653 | 2.1030 | 0.025 * |
| **Sex** | 1 | 0.0903 | 0.02314 | 5.8871 | 0.002 *** |
| **Age** | 1 | 0.1144 | 0.02933 | 7.4611 | 0.001 *** |
| **BMI** | 1 | 0.1639 | 0.04203 | 10.6915 | 0.001 *** |
| **as.character(host_study_id)** | 20 | 2.6739 | 0.68557 | 8.7199 | 0.001 *** |
| **Residual** | 45 | 0.6899 | 0.17690 |  |  |
| **Total** | 71 | 3.9002 | 1 |  |  |

| Supplementary Table 5. Permutational analysis of variance model overall results for unweighted uniFrac matrix | | | | | |
| --- | --- | --- | --- | --- | --- |
|  | **Df** | **SumOfSqs** | **R2** | **F** | **Pr(>F)** |
| **Intervention** | 1 | 0.1172 | 0.0363 | 6.9363 | 0.001 *** |
| **Time** | 2 | 0.0353 | 0.01044 | 1.0451 | 0.396 |
| **Sex** | 1 | 0.1396 | 0.04129 | 8.2649 | 0.001 *** |
| **Age** | 1 | 0.1201 | 0.03551 | 7.1068 | 0.001 *** |
| **BMI** | 1 | 0.1254 | 0.03707 | 7.4196 | 0.001 *** |
| **as.character(host_study_id)** | 20 | 2.0837 | 0.61619 | 6.1664 | 0.001 *** |
| **Residual** | 45 | 0.7603 | 0.22484 |  |  |
| **Total** | 71 | 3.3816 | 1 |  |  |

| Supplementary Table 6. Permutational analysis of variance model overall results for Bray Curtis Dissimilarity | | | | | |
| --- | --- | --- | --- | --- | --- |
|  | **Df** | **SumOfSqs** | **R2** | **F** | **Pr(>F)** |
| **Intervention** | 1 | 0.5246 | 0.03624 | 11.6751 | 0.001 *** |
| **Time** | 2 | 0.1294 | 0.00894 | 1.4395 | 0.066 . |
| **Sex** | 1 | 0.6001 | 0.04145 | 13.3549 | 0.001 *** |
| **Age** | 1 | 0.3956 | 0.02732 | 8.8029 | 0.001 *** |
| **BMI** | 1 | 0.6077 | 0.04198 | 13.5236 | 0.001 *** |
| **as.character(host_study_id)** | 20 | 10.1982 | 0.7044 | 11.3473 | 0.001 *** |
| **Residual** | 45 | 2.0222 | 0.13967 |  |  |
| **Total** | 71 | 14.4778 | 1 |  |  |

| Supplementary Table 7. Permutational analysis of variance model for kombucha group before and after intervention for Bray Curtis Dissimilarity | | | | | |
| --- | --- | --- | --- | --- | --- |
|  | **Df** | **SumOfSqs** | **R2** | **F** | **Pr(>F)** |
| **Time** | 1 | 0.094 | 0.01475 | 1.9679 | 0.025 * |
| **Sex** | 1 | 0.4366 | 0.06853 | 9.1406 | 0.001 *** |
| **Age** | 1 | 0.4206 | 0.06601 | 8.805 | 0.001 *** |
| **BMI** | 1 | 0.3707 | 0.05818 | 7.7602 | 0.001 *** |
| **as.character(host_study_id)** | 13 | 4.3813 | 0.68758 | 7.055 | 0.001 *** |
| **Residual** | 14 | 0.6688 | 0.10496 |  |  |
| **Total** | 31 | 6.3721 | 1 |  |  |

| Supplementary Table 8. Permutational analysis of variance model for kombucha group before and after intervention for weighted uniFrac matrix | | | | | |
| --- | --- | --- | --- | --- | --- |
|  | **Df** | **SumOfSqs** | **R2** | **F** | **Pr(>F)** |
| **Time** | 1 | 0.03781 | 0.02296 | 2.4841 | 0.034 * |
| **Sex** | 1 | 0.06259 | 0.03800 | 4.1122 | 0.007 ** |
| **Age** | 1 | 0.14258 | 0.08656 | 9.3670 | 0.001 *** |
| **BMI** | 1 | 0.05337 | 0.03240 | 3.5062 | 0.008 ** |
| **as.character(host_study_id)** | 13 | 1.13770 | 0.69070 | 5.7494 | 0.001 *** |
| **Residual** | 14 | 0.21310 | 0.12938 |  |  |
| **Total** | 31 | 1.64716 | 1 |  |  |
